# Redundancy Analysis of Capacitance Data of a Coplanar Electrode Array for Fast and Stable Imaging Processing

**DOI:** 10.3390/s18010031

**Published:** 2017-12-24

**Authors:** Yintang Wen, Zhenda Zhang, Yuyan Zhang, Dongtao Sun

**Affiliations:** 1 Institute of Science and Technology, Yanshan University, Qinhuangdao 066004, China; ytwen@ysu.edu.cn; 2 Key Lab of Measurement Technology and Instrumentation of Hebei Province, Qinhuangdao 066004, China; zhangzhenda123@yahoo.com (Z.Z.); dongtaosun@yahoo.com (D.S.); 3 Institute of Electrical Engineering, Yanshan University, Qinhuangdao 066004, China

**Keywords:** coplanar electrode array sensor, redundancy, singular value decomposition, stable imaging, bonding defects, composites material

## Abstract

A coplanar electrode array sensor is established for the imaging of composite-material adhesive-layer defect detection. The sensor is based on the capacitive edge effect, which leads to capacitance data being considerably weak and susceptible to environmental noise. The inverse problem of coplanar array electrical capacitance tomography (C-ECT) is ill-conditioning, in which a small error of capacitance data can seriously affect the quality of reconstructed images. In order to achieve a stable image reconstruction process, a redundancy analysis method for capacitance data is proposed. The proposed method is based on contribution rate and anti-interference capability. According to the redundancy analysis, the capacitance data are divided into valid and invalid data. When the image is reconstructed by valid data, the sensitivity matrix needs to be changed accordingly. In order to evaluate the effectiveness of the sensitivity map, singular value decomposition (SVD) is used. Finally, the two-dimensional (2D) and three-dimensional (3D) images are reconstructed by the Tikhonov regularization method. Through comparison of the reconstructed images of raw capacitance data, the stability of the image reconstruction process can be improved, and the quality of reconstructed images is not degraded. As a result, much invalid data are not collected, and the data acquisition time can also be reduced.

## 1. Introduction

Electrical capacitance tomography (ECT) is a type of imaging technique that has been developed in industrial process tomography applications since the late 1980s and early 1990s [1,2]. In recent years, ECT-based coplanar array electrode sensors have been the object of widespread concern by scholars. Testing using a coplanar electrode sensor is a promising non-destructive testing method that can be used for defect detection in composite materials. This sensor can generate permittivity maps inside an object by measuring the capacitance. This is achieved by systematically applying a potential to an excited electrode and measuring the inter-electrode capacitance between the excited electrode and every other electrode, while ensuring that all the other electrodes are kept at ground [3,4,5]. From the capacitance measurements, an image is then constructed by calculating the distribution of the changes in permittivity [6].

Coplanar array ECT is similar to traditional ECT, which is also ill-posed because the amount of independent capacitance data is far less than the number of permittivity units. To improve this problem, Wei [7], Ren [8] and Ye [9] take the approach of increasing the number of electrodes and change the shape of the sensor. Furthermore, the full incentive pattern is used to get the most out of independent capacitance data [10]. By increasing the number of electrodes, the difference between the capacitive data dimension and the sensitive field dimension is reduced, such that the quality of reconstruction image can be improved to some extent. However, just increasing the capacitance data cannot fundamentally cope with the ill-conditioned inverse problem of coplanar array ECT. It means that a small error in the capacitance data can result in a large number of errors in the solution. The increased data that are acquired from the widely separated electrodes may even be detrimental to achieving a high-quality reconstruction of the permittivity distribution, as well as decreasing the stability of the image reconstruction process. In order to improve the image quality and stability of the image reconstruction process, the redundancy of capacitance data is investigated. 

This paper demonstrates that the stability of the image reconstruction process can be increased only by measuring a subset of all the possible combinations of capacitance measurements without compromising the accuracy of the image reconstruction. In order to facilitate the analysis of capacitance data, the electrode pair has been divided into neighboring, opposing, and distance electrode pairs. The sensitivity map and capacitance measurements are examined using a 3 × 4 coplanar array electrode sensor, and a complete analysis of the capacitance data elucidated.

Here, the factors that might reduce the imaging quality and stability are discussed, that is, the variation of the signal-to-noise ratio (SNR) and the contribution rate of image reconstruction in different electrode pairs. These factors of the coplanar array ECT system are examined by simulation and experiment. On the basis of these results, the measurement reduction method for coplanar array ECT has been defined. Experiments verified that the approach could improve the stability of the image reconstruction process.

## 2. Image Reconstruction Principles

### 2.1. Forward Problem

According to the electromagnetic field theory, any electromagnetic field can be represented by the following Maxwell’s equations:

(1) $$\left\{ \begin{array}{l} \nabla \times H=J_{e}\\ \nabla \times E=-\frac{\partial B}{\partial t}\\ \nabla \cdot B=0\\ \nabla \cdot D=\rho \end{array}\right.,$$

where B is magnetic induction intensity, E is electric field intensity, H is magnetic field intensity, D is potential shift, $J_{e}$ is electric current density, and ρ is charge density. 

For the isotropic medium, the following relationship exists between the electric field and the magnetic field:

(2) $$\left\{ \begin{array}{l} D=\varepsilon E\\ B=\mu H\\ J_{e}=\sigma E \end{array}\right.,$$

where ε is relative permittivity, μ is permeability, and σ is conductivity.

For a capacitive tomography system, the sensor of the capacitive tomography system forms an electrostatic field under application of the excitation. It can be described by the following equation:

(3) ∇⋅(ε∇φ)=−ρ,

where φ is electric potential. If there is no free charge in the electric field (ρ = 0), then the Laplace equation holds:

(4) ∇⋅(ε∇φ)=0,

For Equations (3) and (4), the boundary condition, belongs to the first type of boundary value problem, which is expressed as follows:

(5) $$\varphi {|}_{s}=\mathrm{Const},$$

The positive problem of ECT belongs to the first type of boundary value problem, which can be expressed by the Laplace Equation (4) of the electrostatic field, as follows:

(6) ∇⋅[ε(x,y)∇φ(x,y)]=0,

where ε(x,y) is the relative permittivity distribution of the medium above the sensor and φ(x,y) is the potential distribution.

Taking the 12-electrode system in this paper as an example, when the electrode i is the source electrode (i=1,2,⋯,12), the boundary conditions associated with it is

(7) $$\varphi (x,y)=\left\{ \begin{array}{ll} V_{c} & \;((x,y)\subseteq \Gamma_{i})((x,y)\subseteq \Gamma_{i})\\ 0 & ((x,y)\subseteq \Gamma_{i}(k=1,2,\cdots ,12,k\neq i)\mathrm{and}(x,y)\subseteq (\Gamma_{s}+\Gamma_{\rho g})), \end{array}\right.$$

where $V_{c}$ is boundary excitation voltage, $\Gamma_{1},\Gamma_{2},\cdots ,\Gamma_{12}$ is the surface of the 12 electrodes, $\Gamma_{s}$ is the position of the shield, and $\Gamma_{\rho g}$ is the guard electrode position. 

Calculate the potential distribution according to Equations (6) and (7), and then calculate the induced charge on the electrode plate according to the Gaussian flux theorem, so as to obtain the capacitance between the two electrode plates, namely:

(8) $$C_{ij}=-\frac{1}{V}\oint_{\Gamma }\varepsilon (x,y)\nabla \varphi (x,y)d\Gamma .$$

where V is the potential difference between the two electrodes. After measurement, for an *n*-electrode planar array ECT system, the number of independent measurements is given by m=n(n−1)/2, where n is the number of electrodes in the sensor [10].

Since the relationship between the capacitance measurements and the permittivity of each voxel is nonlinear, the Jacobian matrix is used to linearize the relationship. To establish the relationship between the permittivity distribution and the capacitance measurements, a sensitivity matrix *S* is built according to the following equation [11,12,13,14]:

(9) $$S=\frac{\partial C_{ij}}{\partial \varepsilon }=-\int_{\Omega }E_{i}\cdot E_{j}d\Omega .$$

In the volume integral, $E_{i}$ and $E_{j}$ are the electric field distributions in the entire sensing region Ω when electrodes i and j are excited, respectively. On the left-hand side of the equation, S is the Jacobian matrix, which is also known as the sensitivity matrix. The Jacobian matrix represents change in all of the capacitance measurements when the permittivity has changed, and each row represents the sensitivity of the corresponding capacitance data relative to all the voxels.

The simplified linearized relationship between the change of capacitance and permittivity is given by

(10) ΔC=SΔg,

where vectors $\Delta C\in R^{m}$, ΔC is the change of capacitance, and m is the number of independent capacitance data. On the right-hand side of the equation $\Delta g\in R^{d}$, where Δg is the permittivity change and d is the total number of FEM voxels. $S\in R^{m\times d}$ is the normalized sensitivity map. The map for a certain electrode pair is normalized as

(11) $${S_{pq}}^{*}=\frac{S_{qp}}{\sum_{k=1}^{p}S_{qp}(k)}.$$

In this paper, $\Delta C=C_{\mathrm{full}}-C_{\mathrm{test}}$, where $C_{\mathrm{full}}$ and $C_{\mathrm{test}}$ are the capacitances when measured with the sensor filled with a high-permittivity material and test material, respectively.

### 2.2. Inverse Problem

The inverse problem reflects the permittivity distribution of the cross-section through the measured object by measuring the capacitance value. The inverse problem of the coplanar array ECT process is solved using the Tikhonov based method, which is commonly used to improve the ill-posed issues [15,16]. The main task of image reconstruction is to determine the permittivity distribution from the measured capacitance. In the discrete form, S acts as a constant coefficients matrix in image reconstruction cases [17]. Equation (12) shows the Tikhonov inverse solver:

(12) $$\begin{array}{l} x=S^{T}{\left(S^{T}+aI\right)}^{-1}\Delta C\\ P(x)=\left\{ \begin{array}{lll} 0 & \mathrm{if} & x<0\\ x & \mathrm{if} & 0<x<1\\ 1 & \mathrm{if} & x>1 \end{array}\right. \end{array}$$

where S is derived from Equation (9), ΔC is the change of capacitance, x represents the reconstructed images, α is the regularization parameter, and I is an identity matrix. Ideally, all of the voxels in reconstructed tomogram will have values of 0 when the sensing region is empty. When the sensing region is filled with high-permittivity material, all of the voxels will have values of 1.

## 3. Coplanar Array ECT System

**Figure 1:**
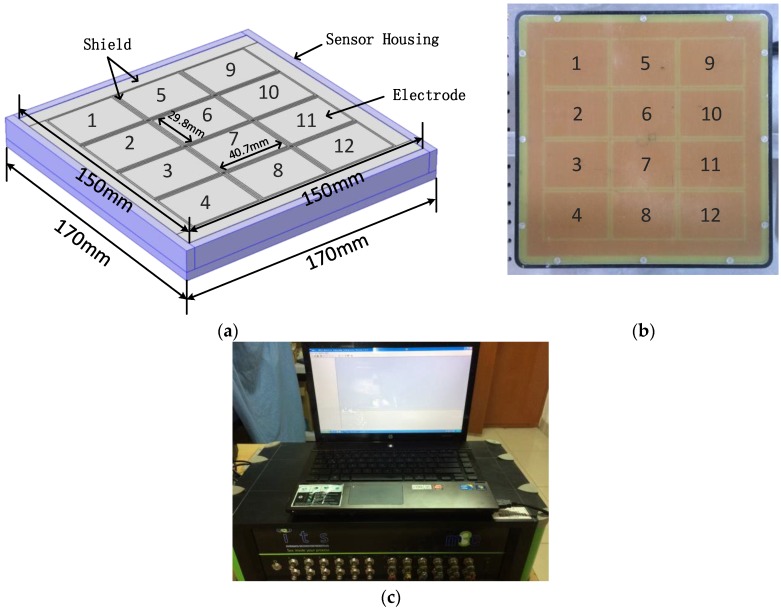
Coplanar array electrode capacitance detection system: (**a**) model of coplanar array electrode sensor; (**b**) planar array 3 × 4 electrode sensor; and, (**c**) Industrial Tomography Systems (ITS) 24-channel electrical capacitance tomography (ECT) M3C system.

The coplanar array ECT sensor used in this work is shown in Figure 1. The sensor includes 12 electrodes that are arranged in a 3 × 4 grid pattern. The sensor is designed accordingly using double-sided printed circuit boards (PCBs). The overall size of the PCB sensor is 170 × 170 mm^2^, and the sensitive area measures 150 × 150 mm^2^. The size of each electrode is 29.8 × 40.7 mm^2^. A grounding conductor surrounds the electrode array to act as a guarding electrode. The bottom layer of the PCB is a pure copper plate that acts as an external shield for the sensor. The planar array capacitance sensor can be protected from external interference by this extra copper layer. Electrodes act as an excited electrode in turn in a typical planar array ECT real measurement process, and the instrument can measure the voltage value between the other electrodes. Therefore, in a typical planar array ECT sensor with 12 electrodes, 66 independent measurements can be made. In order to facilitate the analysis of capacitance data, the electrode pair has been divided into neighboring, opposing, and distance electrode pairs.

For simplicity, in Figure 1b electrode 1 is only taken as an example, with the neighboring electrode pairs including 1–2 and 1–5; the opposing electrode pair, only including 1–6; and, the distance electrode pairs including 1–3, 1–4, 1–7, 1–8, 1–9, 1–10, 1–11, and 1–12. As shown in Figure 1c, the data acquisition system is an Industrial Tomography Systems (ITS) M3C ECT system that can support up to a 24-channel ECT sensor, with an accuracy of 10 fF. The sensor is modeled in ANSYS 12.0 software (ANSYS, Inc., Pittsburgh, PA, USA) to calculate the sensitivity map and simulate capacitance measurements.

## 4. Capacitance Data and Sensitivity Map Analysis

In this section, the results of the analysis of capacitance measurements and the sensitivity map are reported. First, the capacitance measurements were examined from three aspects: (1) the capacitance change in different electrode pairs, (2) the contribution rate of different electrode pairs to image reconstruction, and (3) the SNR of different electrode pairs. Second, the singular value decomposition (SVD) method was used to analyze the reduced sensitivity map.

### 4.1. Capacitance Data

**Figure 2:**
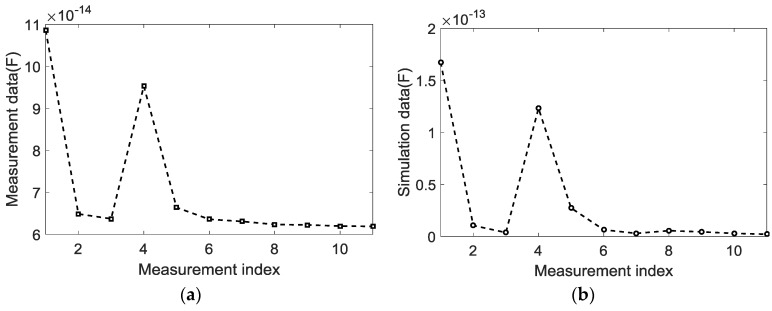
Capacitance data from (**a**) experiment data and (**b**) simulation data.

In this paper, experimental and simulation data were used to compare the relationship between the different electrode pairs in terms of capacitance trends. For simplicity, results are only shown with electrode 1, which was used for excitation, and the remaining 11 electrodes were used for detection. Figure 2 shows the raw capacitance measurements from both simulation and experiment for the coplanar array sensor, which were driven by a sinusoidal signal. By comparing the simulation and experimental data, it can be seen that the trend of the capacitance on each electrode pair is the same, but the experimental data are smaller than the simulation data. The simulated capacitance data is obtained under the ideal condition without parasitic capacitance, which is a set of ideal data. However, in the actual measurement process, as shown in Figure 2a, due to the existence of stray capacitance, the measurement is always the sum of the induced voltage and the stray voltage. Therefore, there is a big difference between the experimental data and the simulation data. The stray voltage is caused by the stray capacitance, which is from the data acquisition system and cables. This stray signal does not correlate with the permittivity distribution; hence, the signal does not contribute much to the image reconstruction.

As shown in Figure 2, the distance between electrodes increased while the capacitance value decreased. The large-capacitance data in both experiment and simulation were found in the 1–2, 1–5, and 1–6 electrode pairs. The largest capacitance was observed in those electrodes in neighboring electrode pairs, 1–2 and 1–5. 

**Figure 3:**
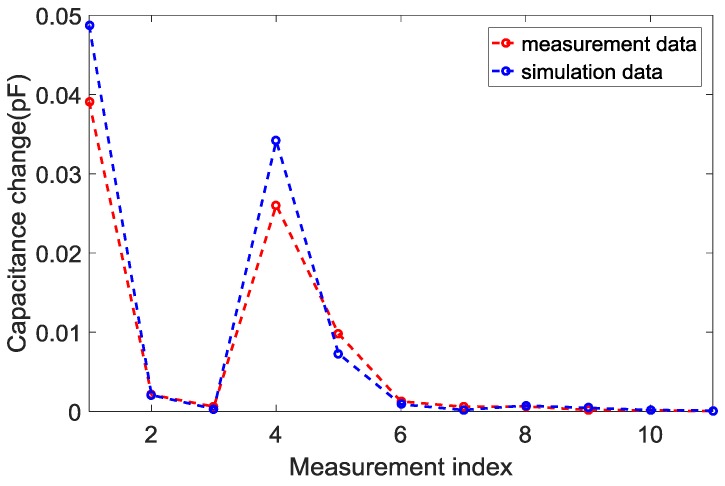
Dynamic range of the capacitance change in electrode 1.

Figure 3 shows the dynamic range of the capacitance change in electrode 1. The capacitance change between the empty (low-permittivity material) and filled (high-permittivity material) permittivity distributions in simulation and experiment showed the same pattern. The large changes in capacitance are found in neighboring electrode pairs and opposing electrode pairs. The small changes in capacitance occurred in the distance electrode pairs.

**Figure 4:**
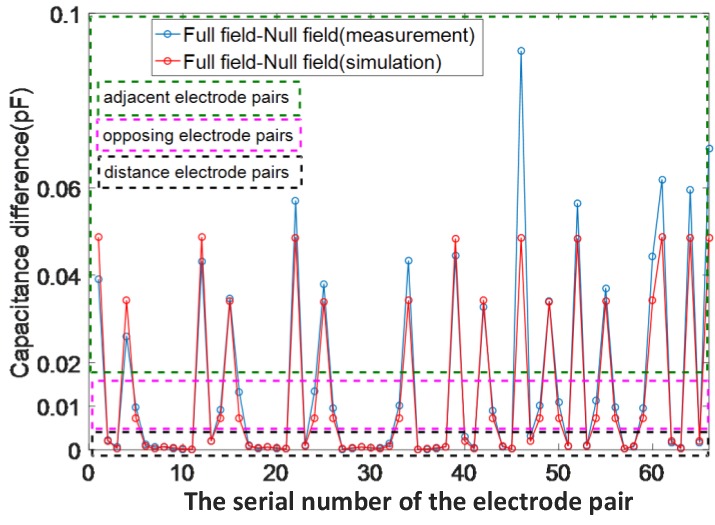
Dynamic range of the capacitance change in total electrodes.

Figure 4 shows the total measurement and simulation capacitance changes. It can be observed in Figure 4 that the regularity of capacitance changes in simulation was similar to that in the experiment. The largest change in capacitance was observed in the neighboring electrode pairs, which was in the range 25–90 fF. The capacitance changes between the opposing electrode pairs were less than those in the neighboring electrode pairs, which were in the range 5–20 fF. For distance electrode pairs that were separated by one or more electrodes, the changes in capacitance were typically in the range 0–2 fF. The capacitance change in distance electrode pairs was less than the minimum measured range of the M3C ECT instrument. Therefore, these measurements provide limited information during image reconstruction.

### 4.2. Contribution Rate to Image Reconstruction

**Figure 5:**
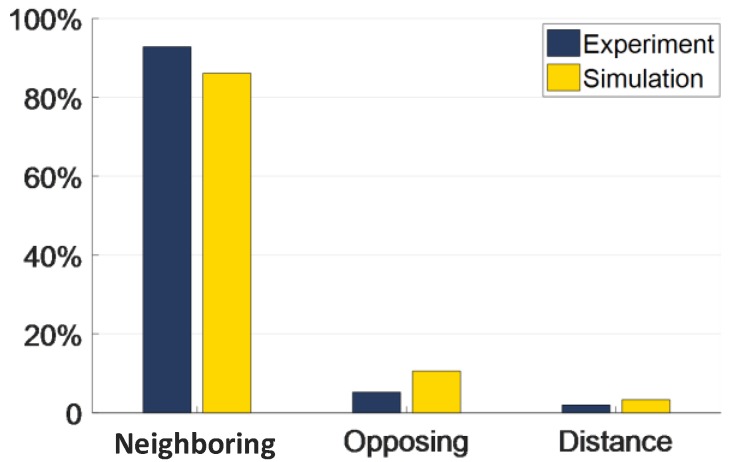
Contribution rate of neighboring, opposing, and distance electrode pairs to image reconstruction.

As shown in Function (10), the greater the variation of capacitance, the greater the contribution rate to reconstructed images. Figure 5 presents the data of the contribution rate to the reconstructed images in different types of electrode pairs in both experiment and simulation. The neighboring electrode pair had the most significant contribution rate to reconstructed images, approximately 90% and 82%, respectively. The opposing electrode pair’s contribution rate was not as important as that of the neighboring electrode pair, which accounted for 5% and 10%, respectively. The distance electrode pair showed a similar pattern when compared to the opposing electrode pair, approximately 1% and 2%, respectively.

### 4.3. SNR of Capacitance Data

**Figure 6:**
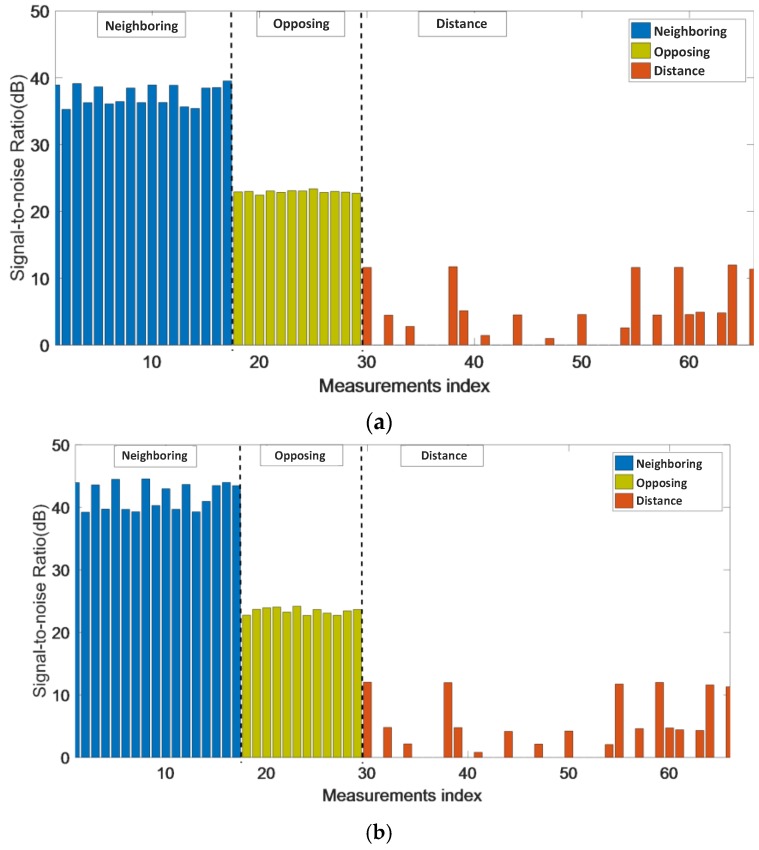
Signal-to-noise ratio (SNR) of experimental and simulation data. (**a**) Experiment (**b**) Simulation.

The inverse problem of coplanar array ECT is ill-conditioned, in which a small error of the capacitance data can lead to a large error in the result. The M3C ECT system can measure the capacitance with an uncertainty (standard deviation) of 1 fF. To approximate the noise in the capacitance data, a 40-dB Gaussian noise of standard deviation 1 fF was added to each capacitance value. The SNRs from different electrode pairs are illustrated in Figure 6. The data from the distance electrode pairs have the poorest SNR, since the capacitance values decrease as the distance between electrodes increases. By comparison, the SNR of the neighboring electrode pair was the largest, approximately 40 and 45 dB, respectively. For the opposing electrode pair, it was not as high as the neighboring electrode pair, nearly 22 dB for both of the electrodes. 

It can be seen in Figure 5 and Figure 6 that the contribution of the distance electrode pairs to the image reconstruction process is minimal and is susceptible to noise. In order to improve the stability of the image reconstruction process, the method of reducing the amount of capacitance data has been proposed. The first data reduction method is used to remove the distance capacitance data and the second to remove the distance and the opposing capacitance data. According to Function (10), after reducing the capacitance data, the sensitivity map needs to be changed accordingly. In order to analyze the reduced sensitivity map, a SVD method is introduced to assess the reduced map.

### 4.4. Sensitivity Map Analysis

**Figure 7:**
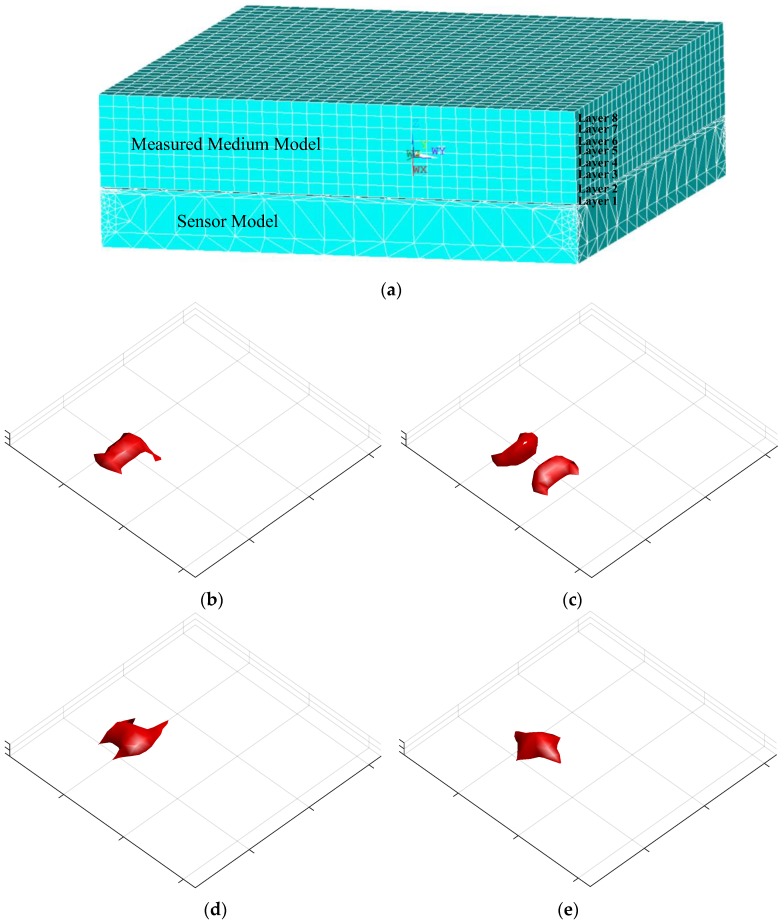
(**a**) FEM mesh model, and the computed sensitivity map between (**b**) electrodes 1 and 2, (**c**) electrodes 1 and 3, (**d**) electrodes 1 and 5, and (**e**) electrodes 1 and 6.

The sensitivity map is analyzed using ANSYS. Figure 7a shows the FEM model of the sensor model and the measured medium model, which are generated by triangular division and mapping division respectively. The measured medium model is equally divided into eight layers, and each layer is divided into 32 × 32 hexahedron elements. Figure 7b,e shows the sensitivity map with different electrode pairs, which were also computed using ANSYS. The sensitive area above the sensor is divided into eight layers and each layer is divided into 1024 hexahedra. The number of elements in this sensitive area is 8192.

According to Function (10), after the amount of capacitance data is decreased, the sensitivity map should also be reduced. Therefore, in order to analyze how image quality varies with the rest of the capacitance data, SVD is used to analyze the sensitivity map.

**Figure 8:**
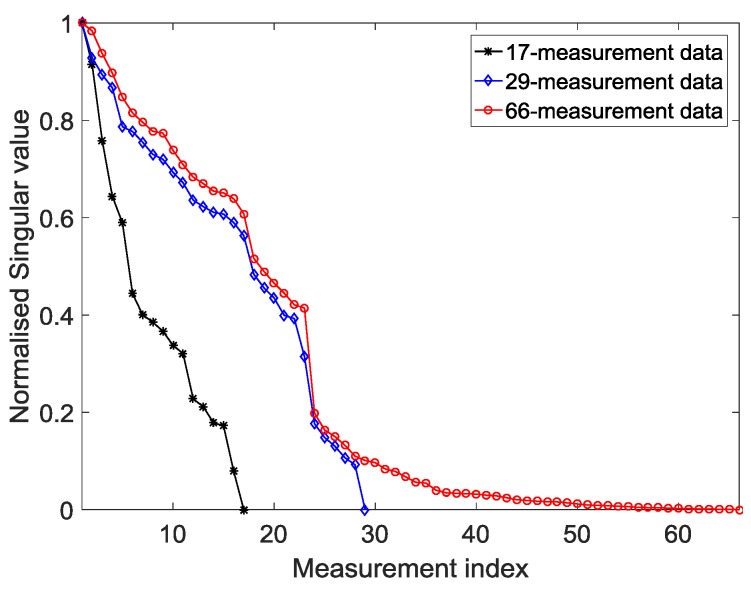
Singular value decomposition. The capacitance data of neighboring electrode pairs comprise 17 groups; that of neighboring and opposing electrode pairs 29 groups; and, that of the original capacitance data 66 groups.

It can be seen from Figure 8 that the gap between the normalized singular value of the 66-measurement capacitance data and that of the 29-measurement capacitance data was narrowed considerably. However, the values of the 17-measurement capacitance data decreased immediately when compared to those of the 66- and 29-measurement capacitance data. Therefore, useful information may be lost when rebuilding the image if 17-measurement capacitance data are retained.

## 5. Analysis of Reconstruction Results

### 5.1. Reduced-Data Image Reconstruction Results

**Figure 9:**
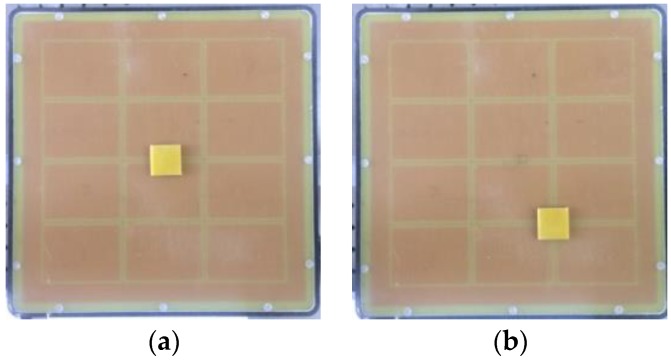

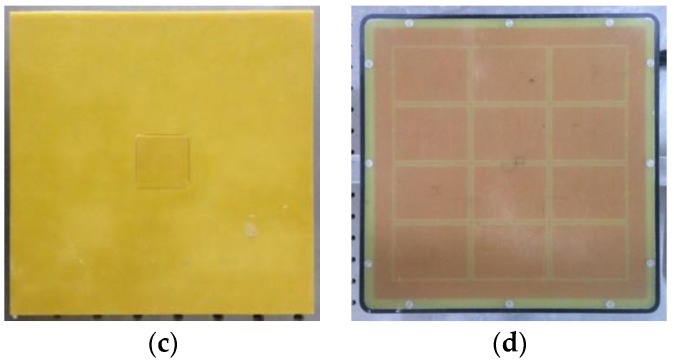
Test samples: (**a**) sample 1, center defect; (**b**) sample 2, edge defect; (**c**) high calibration; and, (**d**) low calibration.

This experiment analyzed the effect of data reduction methods in the image reconstruction process. As shown in Figure 9, two basic types of samples were used in this experiment: samples 1 and 2 are rectangular in shape and are made of all epoxy resin glue, each measuring 15 × 15 mm^2^ on the center and edge of the sensor, respectively. Epoxy resin glue is used for the high calibration and air is used for the low calibration.

**Figure 10:**
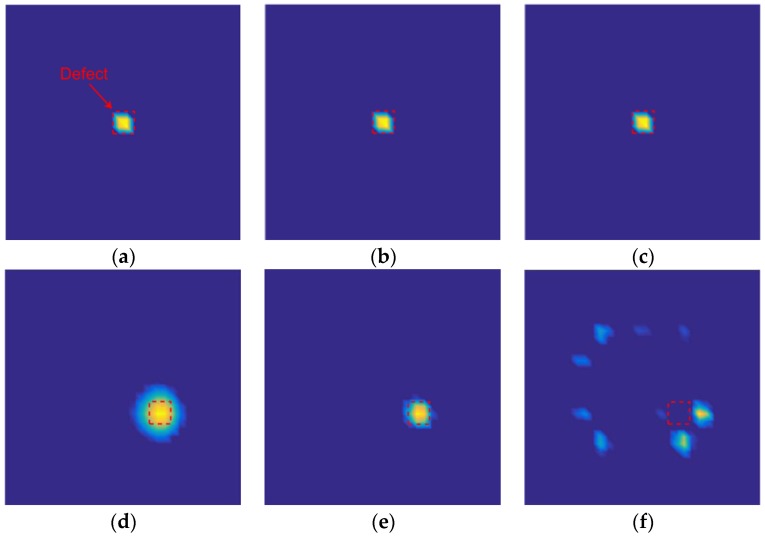
Reconstructed images after data reduction, sample 1 (**a**) 66-measurement capacitance data image reconstruction result, (**b**) 29-measurement capacitance data image reconstruction result, (**c**) 17-measurement capacitance data image reconstruction result, sample 2 (**d**) 66-measurement capacitance data image reconstruction result, (**e**) 29-measurement capacitance data image reconstruction result, and (**f**) 17-measurement capacitance data image reconstruction result.

In this experiment, the fourth image slice was used as the two-dimensional (2D) reconstruction result. Figure 10 shows the permittivity distribution that was obtained both when the amount of capacitance data is reduced and when it is not reduced. As shown in Figure 10a–c, when the defect was on the shield between the two electrodes (Figure 9a), the opposing and distance data were removed from the capacitance data, and the quality of the reconstructed images was not reduced. As shown in Figure 10e,f, when the defect was on the cross positions of four shield (Figure 9b), without opposing electrodes, some defect information was lost. This is the reason that the slope of the 17-measurement capacitance data singular value curve was the steepest one in Figure 8. As shown in Figure 10a,b,d,e, the quality of the reconstructed images was not been reduced after removing the distance data, which explains why the gap between the normalized singular value of the 66- and 29-measurement capacitance data was narrowed considerably.

**Figure 11:**
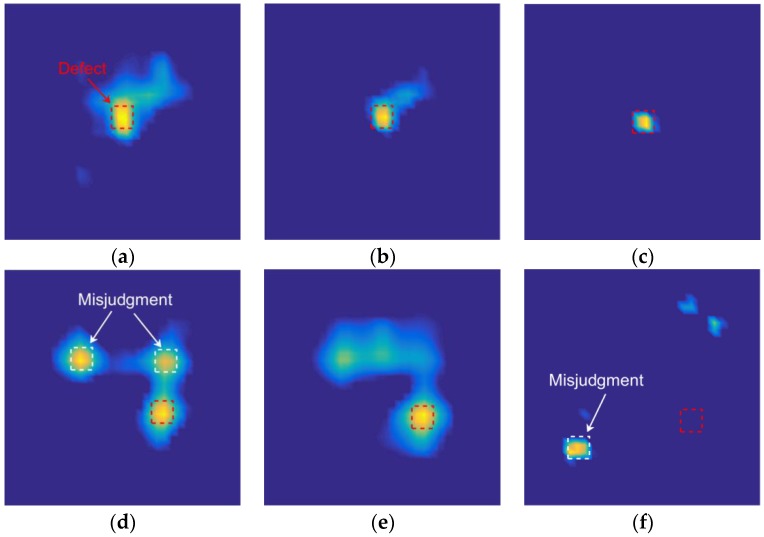
Reconstructed images after data reduction; noise has been added into the capacitance data, sample 1 (**a**) 66-measurement capacitance data image reconstruction result, (**b**) 29-measurement capacitance data image reconstruction result, (**c**) 17-measurement capacitance data image reconstruction result, sample 2 (**d**) 66-measurement capacitance data image reconstruction result, (**e**) 29-measurement capacitance data image reconstruction result, and (**f**) 17-measurement capacitance data image reconstruction result.

Figure 11 shows a similar pattern as Figure 10 because both have the same defect situation. However, the pattern in Figure 10 is slightly different because a 60-dB Gaussian noise was added to the capacitance data prior to image reconstruction. As shown in Figure 11a,d, the images were obtained using full capacitance data, and these data have a significant effect on noise. As shown in Figure 11a,b,d,e, the stability of images has been improved after the removal of the distance data. As shown in Figure 11f, after removing the opposing and distance data, the defect information is lost.

### 5.2. Influence of Material Thickness on Reconstructed Results

**Figure 12:**
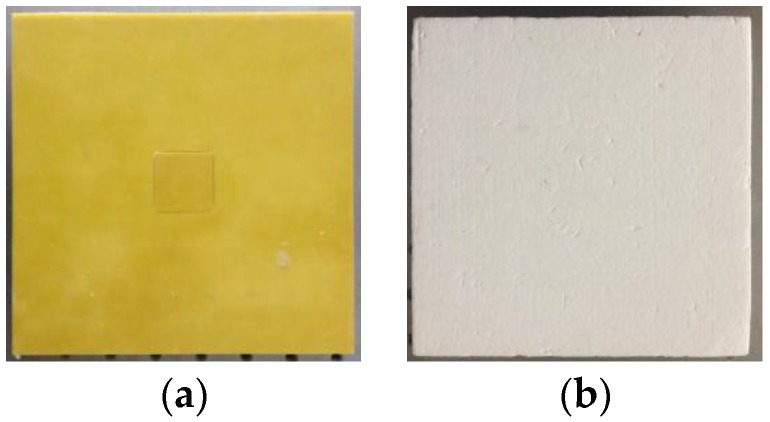

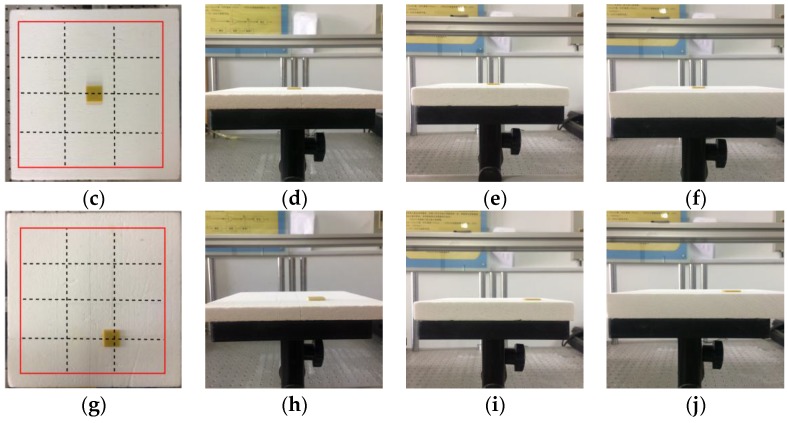
Test samples: (**a**) high calibration; (**b**) low calibration; (**c**) sample 3, center defect; (**d**) sample 4, material thickness 15 mm; (**e**) sample 5, material thickness 20 mm; (**f**) sample 6, material thickness 25 mm; (**g**) sample 7, edge defect; (**h**) sample 8, material thickness 15 mm; (**i**) sample 9, material thickness 20 mm; and, (**j**) sample 10, material thickness 25 mm.

In terms of the defect detection of the composite-material adhesive layer, the composite material’s thickness will greatly affect the signal intensity. Owing to the increase of the material’s thickness, the effective signal may be submerged into the noise, which will reduce the stability of the image reconstruction process. Moreover, the capacitance change is in the range 2–6 fF and is susceptible to noise. Because the effective signal is small, it is easy to be submerged into the noise, which reduces the stability of the image reconstruction process. As shown in Figure 12, a porous ceramic composite (permittivity ~1.3) with thicknesses of 15 mm, 20 mm, and 25 mm is placed above the sensor. The sensor detects the defects in the adhesive layer by penetrating the composite material. The experiment is conducted in a real environment, and the capacitance data is recorded in the absence of noise. Composite material and epoxy resin glue are used for high calibration and composite material without epoxy resin glue is used for low calibration.

**Figure 13:**
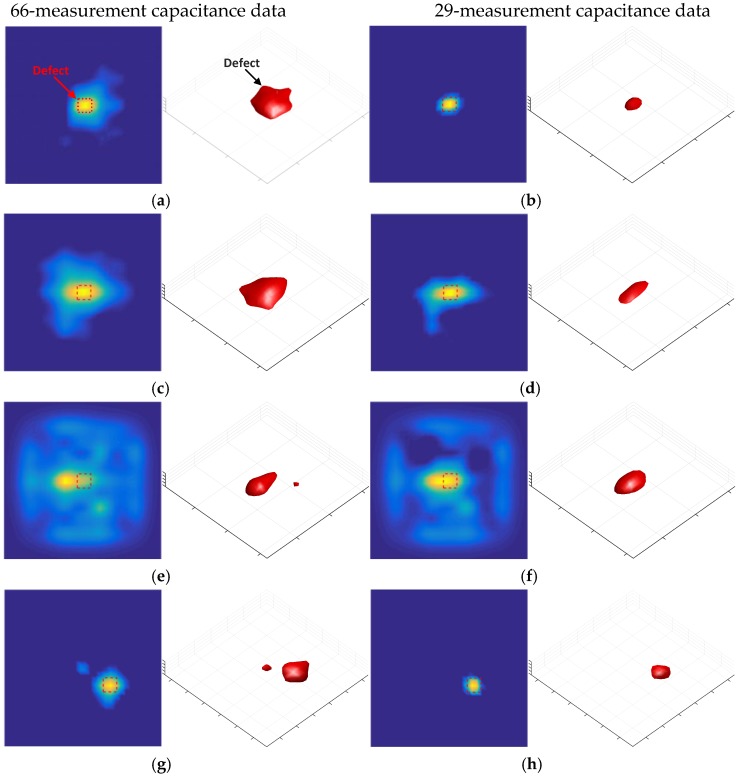

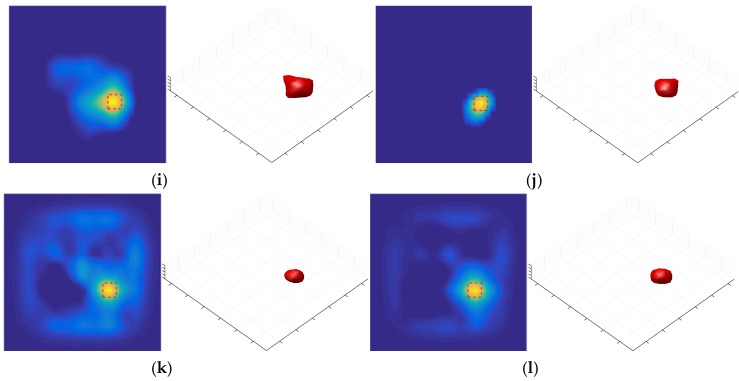
Image reconstruction results in 2D and 3D under conditions of different material thickness, Figure (**a**,**c**,**e**,**g**,**i**,**k**) are 66-measurement capacitance data reconstruction results with thickness of 15 mm, 20 mm, 25 mm; Figure (**b**,**d**,**f**,**h**,**j**,**l**) are 29-measurement capacitance data reconstruction results with thickness of 15 mm, 20 mm, 25 mm. (**a**,**b**) results of sample 4 with thickness of 15 mm; (**c**,**d**) results of sample 5 with thickness of 20 mm; (**e**,**f**) results of sample 6 with thickness of 25 mm; (**g**,**h**) results of sample 8 with thickness of 15 mm; (**i**,**j**) results of sample 9 with thickness of 20 mm; (**k**,**l**) results of sample 4 with thickness of 25 mm.

In this experiment, the fourth image slice is used as the 2D reconstruction result and the three-dimensional (3D) image is a superposition of all the slices. It can be seen from Figure 13 that as the thickness of the material increased, the capacitance data were aggravated by noise interference, which increased the edge blur of the reconstructed image. As shown in Figure 13, after removing the distance electrode data, the edge blurring of the reconstructed image was weakened and the contour of the defect was clear. The experimental results suggest that the capacitance data from distance electrode pairs in a coplanar array ECT sensor are not beneficial, and may even be detrimental to achieving stable and high-quality image reconstruction. Removing the distance electrode can improve the stability of the reconstruction process, while reducing the data acquisition time.

## 6. Conclusions

In this paper, capacitance data redundancy analysis method for composite-material adhesive-layer defect detection has been presented. In a 3 × 4 coplanar array ECT measurement, one capacitance value is acquired for each pair of electrodes in the sensor, which can be time consuming if a large number of electrodes are used. Moreover, some of the measurements from distance electrode pairs may make the inverse problem more challenging, and they cannot provide useful or robust measurements. 37 measurements from distance electrode pairs can be made using this sensor. Therefore, the ill-conditioning of the inverse problem will be aggravated by using these unstable data. The increase of material thickness aggravates the noise, and the hazard becomes even more pronounced. In this study, this article has provided proof of data reduction that can improve the stability of the image reconstruction process, while preserving image quality. 

The data redundancy analysis is based on the contribution rate of image reconstruction and on the anti-jamming capability of different electrode pairs. The reduced sensitivity map was evaluated with SVD. After removing the useless data, the image can only be reconstructed by using neighboring and opposing electrode measurements (29 measurements). As a result, the stability of the image reconstruction process has been improved and the data collection time reduced. The experimental results are valuable and the data reduction method can potentially be an effective method for data processing.

The data redundancy analysis method proposed in this paper is also relevant in designing suitable arrangements of electrodes in coplanar array sensors. This paper has demonstrated that the neighboring and opposing measurements have the most significant contribution to image reconstruction and are more robust. Therefore, in sensor design the appropriate number of electrodes should be selected in order to make the proportion of effective capacitance data the largest. When a sensor contains an appropriate number of electrodes, the capacitance data have strong anti-interference ability and the imaging process is more stable.
